# Next Generation Delivery System for Proteins and Genes of Therapeutic Purpose: Why and How?

**DOI:** 10.1155/2014/327950

**Published:** 2014-07-15

**Authors:** Ashish Ranjan Sharma, Shyamal Kumar Kundu, Ju-Suk Nam, Garima Sharma, C. George Priya Doss, Sang-Soo Lee, Chiranjib Chakraborty

**Affiliations:** ^1^Institute for Skeletal Aging & Orthopedic Surgery, Hallym University Chuncheon Sacred Heart Hospital, Chuncheon 200704, Republic of Korea; ^2^Department of Physics, School of Basic and Applied Sciences, Galgotias University, Greater Noida 203201, India; ^3^Medical Biotechnology Division, School of Biosciences and Technology, VIT University, Vellore, Tamil Nadu 632014, India; ^4^Department of Bioinformatics, School of Computer Sciences, Galgotias University, Greater Noida 203201, India

## Abstract

Proteins and genes of therapeutic interests in conjunction with different delivery systems are growing towards new heights. “Next generation delivery systems” may provide more efficient platform for delivery of proteins and genes. In the present review, snapshots about the benefits of proteins or gene therapy, general procedures for therapeutic protein or gene delivery system, and different next generation delivery system such as liposome, PEGylation, HESylation, and nanoparticle based delivery have been depicted with their detailed explanation.

## 1. Introduction 

Over the last few years, numerous therapeutic proteins and peptides have been approved for clinical usage. Till date, more than 135 different therapeutic proteins and genes have been approved by US-FDA for clinical use, and various therapeutic proteins are in the process of development [1, 2]. It was a landmark discovery in the medical science when insulin was purified from bovine and porcine pancreas and was utilized as a life-saving injection for patients with type I diabetes mellitus (T1DM) in 1922 [3]. At that time, some issues were associated with this insulin treatment such as availability of animal pancreases especially bovine and porcine pancreases, immunogenicity of animal insulin to some patients, and cost of the protein [4]. It was noted that about 5% of all patients were having insulin allergy [5]. The problem was solved through recombinant DNA technology, which helped in the production of recombinant insulin using* E. coli* expression system [6, 7]. Insulin was the first commercially available recombinant therapeutic protein, approved by the US-FDA in 1982, and presently is the most significant treatment for T1DM [8, 9]. Presently, with the help of biotechnology and recombinant DNA technology, several recombinant therapeutic proteins are being developed and marketed as biopharmaceutical, and the sales value of these recombinant proteins has gained the highest level of market share in pharmaceutical sector [10, 11].

With the beginning of recombinant DNA technology, the idea was to use nucleic acids to cure diseased cells, especially in cells where gene is deleted or mutated. For this mode of therapeutic application, in 1972, Friedmann and Roblin gave the term “gene therapy” [12]. After this report, there have been many debates on pros and cons of gene therapy technology [13]. However, slowly, due to novel advantages of gene therapy, it is entering into the mainstream of treatment. More than 1800 gene therapy clinical trials have been completed throughout the world and many are continuing [14]. Therefore, developing efficient gene delivery technology is one of the significant areas for pharmaceutical industry in current era [15].

Presently, pharmaceutical delivery system (PDS) or drug delivery system (DDT) is very important for the pharmaceutical industry. Many pharmacological properties of traditional molecules can be improved with the help of DDS [16, 17]. The effectiveness and marketability of the drug molecules depend on the mode of DDS. Pharmaceutical industries are prone to generate new DDS which can impart novel properties to existing as well as newly discovered products. New DDS will be more efficient and safer compared to the existing one [18]. Presently, many existing drug molecule/marketed drugs use new delivery systems and are of great interest for doctors or medical professionals [19, 20]. It has been noted that market value, competitiveness, and patent life may boost up for an existing drug candidate molecule if we use a new DDS. Therefore, the existing drug candidate molecules may offer a new opportunity to increase the market price and competitiveness in the pharmaceutical market [21]. Conversely, patent expiry is one of the major alarms for the pharmaceutical industry. A new DDS can provide a new marketability to an existing drug molecule. Therefore, the development of novel delivery systems is at high priority for the pharmaceutical companies to capture global market. Pharmaceutical market is projected to have a growth with compound annual growth rate of approximately 5% [22]. Biopharmaceuticals (especially therapeutic proteins and gene therapy) are one of the fastest growing areas of the pharmaceutical business. The first generation therapeutic protein based drugs are currently passing through a number of difficulties and needs for improvement. The therapeutic protein delivery system (TPDS) offers longer circulation time for the therapeutic protein in the patient's body and enhanced pharmacokinetics (PK) and pharmacodynamics (PD) properties and is now extremely valuable from the commercial point of view [23]. One the other hand, the efficient gene delivery system can improve the means for delivering genes during gene therapy and thus can contribute toward more successful clinical outcomes [24].

In this paper, we have tried to highlight next generation delivery systems and benefits of proteins therapy or gene therapy. Efforts have been made to summarize general procedures for therapeutic protein or gene delivery system and different next generation delivery systems, namely, liposome, PEGylation, HESylation, and nanoparticle based delivery along with their detailed description.

## 2. Why Proteins Therapy or Gene Therapy?

Over the last few years, biopharmaceuticals especially therapeutic proteins have received great attention. As per the research and markets report by “Global Protein Therapeutics Market Forecast to 2015,” the global market for biopharmaceuticals is growing and is likely to reach the target of $143.4 by 2016. Among the biopharmaceuticals, therapeutic proteins and genes delivery have gained the maximum percentage of market share [25].

It has been found that protein therapeutics has some advantages over small-molecule drug molecules, which may be summarized as follows. (i) Therapeutic proteins can provide efficient replacement treatment when gene is deleted or mutated. This treatment can help us without any gene therapy. (ii) Proteins perform very scrupulous and multifarious functions which are explicit and exclusive. It is very difficult to imitate this distinctive possessed function of enzymes by simple chemicals. (iii) It has been noted that the effect of proteins is extremely specific. So, there is very little chance for the hindrance of normal biological processes with the therapeutic proteins that cause unsympathetic effect. (iv) Biologically, our body creates many kinds of proteins which can be used as therapeutics. Since these proteins are produced from our body itself, they are well tolerated. Therefore, the chance of failure is fewer during the clinical trials. (v) The regulatory approval time of therapeutic proteins is faster than that of small-molecule drugs. The regulatory authority in USA, US-FDA, approves a therapeutic protein compared to small-molecule drugs in the short span of time. From financial point of view, these benefits make therapeutic protein attractive to the pharmaceutical industry [1, 26].

Gene therapy may provide novel treatments for diseases having no effective conventional treatment. Gene therapy can be the ultimate solution for genetic disorders, as it can help to replace deleted or mutated gene for correcting genetic disorders. This possibility of amending genetic disorder is gaining importance and researches are trying to deliver genes to the affected cells. Major factor affecting efficacy for gene therapy is gene delivery system. The refinements to the delivery system may increase security as well as the long-term expression of the gene of interest and reduce the chance of mutagenesis of the particular gene. After gene replacement therapy, the patient needs not receive the treatment of protein based therapeutics regularly, making it one of the desired lines of treatment [27, 28].

## 3. General Strategies for Therapeutic Protein or Gene Delivery System 

Other than the above benefits, some limitations have been noted of therapeutic proteins and genes. The main disadvantage is the stability associated with these proteins or genes which is often not proper. The half-life is also limited. Immunogenicity is another problem for therapeutic protein or genes. For the therapeutic proteins, it has also been observed that light sensitivity, moisture, temperature, and so forth, hamper their stability. Many strategies have been undertaken to improve these limitations. Among them, two strategies are frequently being employed: one is the change in the therapeutic protein (development through the alteration in protein configuration or covalent add-on) itself and through development in the formulation [29, 30]. Proteins are generally conjugated with natural or synthetic polymers (PEGylation, HESylation, and polysialylation) to alter structure of therapeutic proteins [31, 32]. Conversely, different drug formulation systems are also being used to overcome the existing limitations of therapeutic proteins. These formulation systems are polymeric microspheres, polymeric nanoparticles, liposomes, and so forth [33].

For gene delivery, viral vectors and nonviral vectors are usually used. Major viral based gene delivery systems are adeno-associated viral vectors [34]; retroviral/lentiviral vectors [35] and nonviral based delivery systems are cationic liposome [36] and PEGylated system [37].

### 3.1. Liposome for Therapeutic Protein or Gene Delivery System

The efficiency of a number of drugs is often limited by their potential to reach the site of therapeutic effect. In most cases, only a small amount of a controlled dose reaches the target site, while the majority of the drug allocates throughout the rest of the body in accordance with its physic-chemical and biochemical properties. Therefore, it is very challenging task to enhance the pharmaceutical effect of drugs while reducing its toxicity* in vivo*. These objectives can only be achieved through next generation delivery system. Lipid molecules of biomembranes interacting with water molecules can control the transport phenomena and protein functions with anisotropic flow experience. After the discovery in 1965, liposomes were used for delivery of peptide and protein drugs [38–41]. For the development of liposome-based drug delivery system, a consistent size distribution is necessary to produce the nanocarrier's* in vitro *features (e.g., drug loading capacity, aggregation, sedimentation, etc. [42, 43]). Considerable attention has been paid for liposomal drug delivery systems due to their specific attributes, such as (i) successful encapsulation of molecules where both tiny and large molecules are present and the molecules are having a wide range of hydrophobic levels and pKa values; (ii) prolonging and target release of therapeutic agents by modification of liposome surface; and (iii) minimization of clinical drug dose and reducing toxicity results [44, 45].

**Figure 1:**
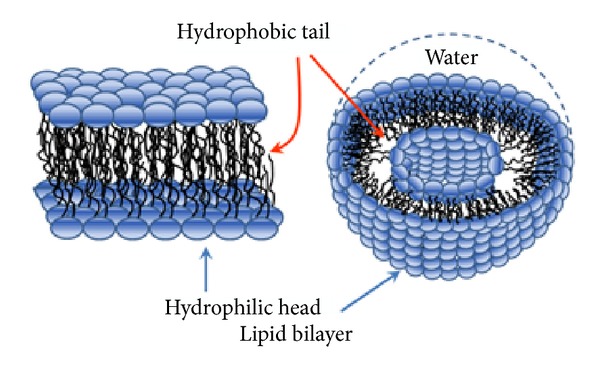
Diagrammatic representation of lipid bilayer used for encapsulating therapeutic proteins and genes for their delivery.

A number of experimental reports have been successfully published on the medical use of liposomes, consisting of the lipid bilayer membrane, as a drug carrier for the purpose of the reduction of drug toxicity or targeting of drugs to specific cells [46–49] (Figure 1). Clearly, it is not probable to deal with all relevant issues, so emphasis will be made to address some key topics, including successes and main challenge and limits of liposomes in protein and peptide delivery.

### 3.2. Liposome Preparation

The main objective for the use of liposome as drug carriers is to target specific tissues such as tumours and also to reduce toxic side effects in sensitive organs such as liver, heart, and kidneys. Additionally, it is possible to extend the therapeutic index of liposomal carriers over that of the corresponding conventional formulations by optimizing the lipid composition, liposomal size, membrane fluidity, surface charge, steric stabilization, and so forth.

The amphiphilic molecules used for liposomal preparations are based on the structure of biological membranes lipids [57–63]. For liposome synthesis two hydrocarbon chains are usually esterified to a glycerol backbone. These hydrophobic chains are further connected to a hydrophilic head group containing either a phosphate or some carbohydrate units. These lipid head groups are either zwitterionic (phosphatidylcholine, phosphatidylethanolamine, sphingomyelin), negatively charged lipids (phosphatidic acid, phosphatidyl glycerol, phosphatidyl serine, phosphatidyl inositol, cardiolipin, substituted glycolipids such as monosialoganglioside), or entirely uncharged lipids (unsubstituted glycolipids). Examples of cationic amphiphiles are DOTAP, DODAC, DC-Chol, DMRIE, DOTMA, DOSPA, DOGS, and many others.

Amphiphilic lipid monomers are weakly soluble in water having low critical micelle concentration (CMC), depending on the hydrocarbon chain length. These single-chain lipids (lysolipids, free unsaturated acyl chains, detergents, etc.) spontaneously assemble into micelles which further act as membrane lipids and tend to form bilayers. Figure 1 illustrates the bilayer structures which form closed vesicles, that is, liposomes. One can distinguish between multilamellar and unilamellar vesicles which can be varied from minute vesicles (size, <100 nm), large vesicles (size, 100–500 nm), or huge vesicles (size, ≥1 *μ*m). Some isolated lipids or lipid mixtures may prefer nonbilayer morphologies such as hexagonal and cubic phases.

Therapeutic genes and proteins can be (i) encapsulated within the liposome and (ii) chemically conjugated to the surface groups. With the help of liposome, passive encapsulation can be achieved by incubating genes, protein, or peptide at or somewhat lower than the phase transition temperature, used for the preparation of liposome. Vigorous loading of therapeutic genes and proteins, termed as triggered loading, can also be achieved by increasing temperature in presence of ethanolic buffer and mild swirling for a particular period. This simple process is somewhat fast and is used to attain higher encapsulation efficiency [64]. Usually proteins are required to exist in aqueous core position. On the other hand, uncovered hydrophobic regions of protein may work together with the lipid membrane. However, the interaction between proteins and lipids are normally to maintain the bioactivity of proteins [65].

Initially, conjugation of proteins with the liposomes was explored by means of glutaraldehyde or 1-ethyl-3-(3-dimethylaminopropyl) carbodiimide (EDC); afterwards researchers are also working on selective bi-functional coupling agents [66, 67]. These reactions encouraged the development of liposome into additional advanced forms and include (i) immunoliposomes, conjugated to antibodies or antibody fragments [68, 69], (ii) stealth liposomes connected with PEG, provides protective coat for evading recognition by opsonins and slowing down clearance [70–72], (iii) extended flowing immunoliposomes coated together with protecting polymer and also with antibodies [71, 73], and (iv) the next generation of liposomes which permit alteration to the exterior surface through a number of compounds that are either alone or in concert including stimuli sensitive lipids, polymers, cell penetrating peptides, and diagnostic agents [72, 73].

For the treatment of liver tumours or metastases, investigators are continuing to use galactosylated liposome for targeted delivery of drugs to liver [74]. The capability of these galactosylated liposomes led to their use in gene delivery systems to deliver in targeted cells [75]. The presence of lipid that is able to form nonbilayer structures, such as dioleoylphosphatidyl ethanolamine (DOPE), can endorse destabilization of the bilayer, inducing fusion events. DOPE has been particularly beneficial for cationic liposomes complex formation with plasmid DNA for gene delivery [76, 77].

### 3.3. Liposomes Acting as Carriers of Protein and Gene Therapy

Biologically active complexes of genes and proteins, for example, small interfering RNA (siRNA), cytokines, enzymes, peptide hormones, and others, are the choice of drugs which could be very useful for the treatment of various diseases. The incorporation of these therapeutic moieties/drugs in liposomal membranes offers several advantages such as high drug incorporation efficiency; stable confinement of drugs in the liposome; prevention of drugs against metabolic degeneration; and long-term therapeutic stage. The supportive effects provided by liposomes have been employed to a wide range of proteins and genes. Superoxide dismutase (SOD), a cytotoxic agent used during phagocytosis, is an enzyme which protects from the effects of superoxide anion. Liposomal encapsulation of SOD has been found to increase its performance, extend circulation, and reduced membrane peroxidation in different areas of brain [78, 79]. Spray-dried powder formulations of the active SOD in liposomes mixed with disaccharides have also been described [80]. The potential ability of liposome-encapsulated enzymes to enter the cytoplasm or lysosomes of live cells is of crucial importance for the treatment of congenital diseases produced by the abnormal behaviour of some intracellular enzymes [81]. Gaspar et al. reported that survival of animals with asparagine dependent tumours associated with free enzymes is increased by the application of liposome-encapsulated asparaginase [82]. In addition, such liposomal encapsulated asparaginase also avoids the formation of anti-asparaginase antibodies. In another study, enhanced thrombolytic activity was observed by tissue plasminogen activator encapsulated in liposomes, as compared to native enzyme, when employed for thrombolytic treatment in rabbits with jugular vein thrombosis [83]. An interesting approach applying liposome liposomes encapsulated enzymes is antibody-directed enzyme prodrug therapy (ADEPT), based on the on-site activation of chemically modified inactive anticancer and antiviral prodrugs into active therapeutic agents [84]. To achieve the specific production of active cytotoxic molecules from inactive drugs in the areas of tumour cells, a conjugate drug was developed using tumour-specific antibody along with an enzyme responsible for the conversion of inactive drug into the active form. For enhancing the enzymatic activity of obligatory enzyme in tumour cells, moderately than just “straight” antibody-enzyme conjugates, a unique liposome, namely, immunoliposomes, is loaded with the essential enzyme [85].

In spite of intensive efforts intended for designing a number of different cationic lipids [86–88], gene expression can only be detected after local administration instead of systemic injection, along with the evident toxic side effects of cationic lipids [89, 90]. Cationic lipid-DNA complexes face supplementary issues due to their large size and high surface charge combining together to result in fast elimination from the circulation. However, large numbers of theories are emerging from huge and quickly rising literature in the arena of delivering nucleic acids which are (i) positively charged cationic lipids, which is considered necessary for the effective relationship of nucleic acids with lipids [91], (ii) liposomes with positive charge results in their fast clearance by the mononuclear phagocyte system (MPS) and not specific cell binding [92], (iii) the circulatory half-life of liposome mediated delivery of nucleic acids that can be increased by modifying surface charge to near neutrality either by coating the cationic liposomes (CCLs) [93] or by using of ionizable lipids [94–97], (iv) for particular binding and internalization, the targeted ligands being mandatory [98, 99], and (v) efficient endosomal release following internalization being needed for therapeutic activity [100], which can be provided by ionizable cationic lipids with optimized bilayer destabilizing capacities and pKa [97, 101].

## 4. PEGylation Carriers of Therapeutic Proteins and Genes 

PEGylation is a process through which polyethylene glycol (PEG) chains are conjugated to proteins (therapeutic proteins), peptides, or any molecules. In 1990, US-FDA approved the first PEGylated therapeutic protein and its brand name is Adagen (pegadamase), marketed by a USA pharma company (Enzon Pharmaceuticals) for the cure of Severe Combined Immunodeficiency Disease (SCID) [102]. After that, US-FDA approved about seven therapeutic proteins [103]. Till date, several therapeutics (approximately 80 polypeptide medicines) are marketed in USA and approximately 350 are undergoing clinical trials. Among them, many are PEGylated therapeutic protein [104]. Through the PEGylation process, the molecular mass of therapeutic proteins is increased. Therefore, it guards the therapeutic protein from the proteolytic enzymes and thereby degradation of the proteins. It has been noted that PEGylation process improves pharmacokinetics of the therapeutic protein.

### 4.1. Procedure of PEGylation

PEG is hydrophilic, safe, nonimmunogenic polymers. These polymers are chemically inert repetitive units of ethylene oxide. In the toxic point of view, this molecule is generally accepted as safer molecule [103]. PEG reagents are commercially available as linear or branched configurations with different lengths, shapes, and chemistries and molecular weights. It is commercially available from some companies from Asia, particularly such as NOF corporation (Japan); SunBio (South Korea); Reddy's Lab (India), and JenKem (China). Some other important companies are Chirotech Technology Limited (UK), Creative PEGWorks (USA), and so forth [104].

It is compulsory to activate the PEG moiety to conjugate it with therapeutic proteins. For the reaction with PEG moiety, different chemical groups in the amino acid of therapeutic protein side chains such as NH2, –NH–, –COOH, –OH, –SH groups and disulfide (–S–S–) bonds can be exploited. Therefore, in this process, reaction occurs between the amino acid of therapeutic protein and suitably activated PEGylation reagents. It has been shown that the reactive amino acids that often participate during this conjugation process are arginine, aspartic acid, histidine, lysine, cysteine, glutamic acid, threonine, tyrosine, and serine. Other than that, N-terminal amino group and the C-terminal carboxylic acid are also found to be involved with these reactions [105].

**Figure 2:**
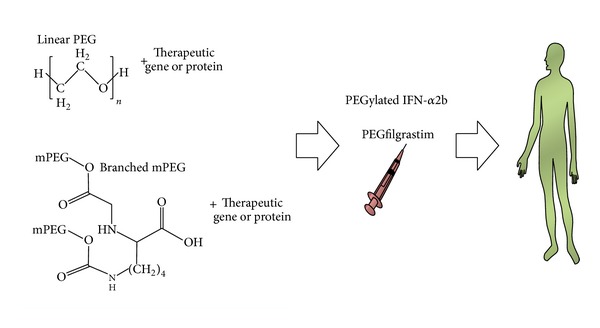
Schematic diagram representing systemic delivery of therapeutic proteins or genes following conjugation with polyethylene glycol molecules. Here, structural formulae for linear PEG and branched mPEG are also displayed.

Several PEGylated therapeutic proteins have been reported till date, which includes Peginterferon *α*2b (PegIntron), PEGylation of IFN-*α*2a as a preliminary therapy for the chronic hepatitis C [106, 107], and mono-PEGylated TNF-*α* for antitumor treatment [108] (Figure 2).

Some PEGylated gene therapy has also been performed. Adenosine deaminase-deficiency (ADA-SCID) is a kind of immunodeficiency. ADA is involved in the purine salvage pathway and absence of this enzyme leads to build-up of intracellular and extracellular substrates (adenosine or deoxyadenosine) leading to adverse effects on the functions of different cell types. In case of immune cells it leads to severe lymphopenia with abnormal development of T, B, and natural killer (NK) cells. In order to cure this immune disorder, PEGylated adenosine deaminase gene has been transferred to T lymphocytes [109, 110]. Gene therapy for ADA-SCID shows great promise in the treatment of this disease. Using this delivery system, approximately 30 patients with ADA-SCID have been treated worldwide [111, 112]. It has been reported that immune function has been regained without the support of enzyme replacement therapy [113]. Moreover, there were no adverse events reported related to the PEG gene transfer technology [114, 115].

### 4.2. Advantage of PEGylation Procedure

This process augments the solubility of therapeutic proteins. It provides solubility to different solvents such as water and various organic solvents. It has been witnessed that the PEGylated therapeutic protein enhances property for site specific performance. It has also been found to enhance PD, PK properties of the protein. Conversely, this procedure diminishes immunogenicity [116].

## 5. HESylation

HESylation utilizes a hydroxyethyl starch derivative for conjugation to proteins (therapeutic proteins) or drug molecules to increase its size. HESylation name has been derived from “HES” which corresponds to a part of hydroxyethyl starch derivative. HES are natural polymers present in starch along with amylopectin fibers. HES are produced from natural maize starch. Therefore, they are highly biocompatible and biodegradable and are clinically approved as plasma volume expanders (PVEs). These attributes make it an attractive hydrophilic polymer for half-life extension (HLE) technologies [117, 118]. HESylation delivery system provides extended circulation half-life to the therapeutic molecules. It has been observed that it increases the stability of therapeutic protein and amplifies biological activity. A European pharma company (Fresenius Kabi, www.fresenius-kabi.com) is regularly applying HESylation delivery system to a variety of proteins (e.g., Erythropoietin (EPO) and Granulocyte-colony stimulating factor (G-CSH)) [26, 119].

## 6. Nanoparticle Based Delivery 

**Figure 3:**
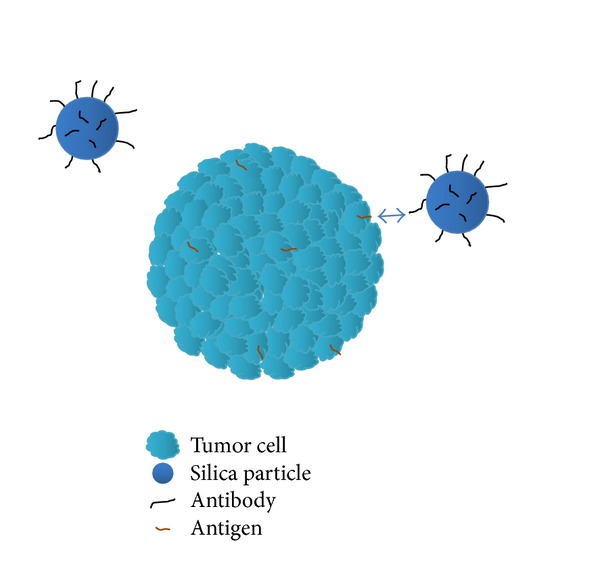
Schematic diagram depicting targeted delivery of antibody labelled silica nanoparticle to the tumour cell antigen.

Nanoparticle based delivery of therapeutic proteins and genes is believed to the significant area of drug delivery (Figure 3). For delivery of therapeutic protein or drug, a number of protein-nanoparticle based deliver systems are being used such as albumin [120], gelatin [121], and legumin [122]. Conversely, many natural polymers and their derivatives like chitosan, dextran, and starch nanoparticles have also been tried to deliver different proteins and genes.

It has been recently documented that dendrimers [123] biodegradable polymeric nanoparticles [124] and gold nanoparticles [125] have been used for gene therapy. Researchers are usually exploiting two techniques for nucleic acids delivery, that is, encapsulation or conjugation. For nucleic acids like plasmid DNA, RNA, and siRNA, encapsulation methods are usually preferred to deliver nucleic acids with nanoparticles [126]. However, sometimes these nucleic acids are also being conjugated with the nanoparticle for delivery [127–130]. One of the methods to link nucleic acids to a nanoparticle is to modify the surface of the nanoparticle and to provide a positive charge. Positive charge on nanoparticle would favour easy binding of negatively charged DNA. However, this method is used for liposome and other polymer-mediated gene transfer [131]. Recently, some researchers have generated polycationic amphiphilic cyclodextrin-based nanoparticles [132] and it has been employed for gene delivery of interleukin-12 (IL-12). For siRNA therapeutic delivery, one group of researchers used arginine-engrafted biodegradable polymer as delivery system [133]. This delivery system improved accumulation of carrier-siRNA complexes in the tumour tissue. However, there is vital need for the production of a common platform for nanoparticle based delivery systems which can be customized only to deliver different kinds of nucleic acids such as DNA, RNA, and siRNA without any side effect to the patients.

## 7. Future Prospects

Delivery systems for proteins and genes have taken more than 25 years to emerge as a feasible pharmaceutical tool and several therapeutic proteins and genes are marketed already (Table 1). Liposomes, PEGylation, HESylation, and nanoparticle based delivery are now established as the processes of choice for improving the PK and PD of protein and gene based therapeutic pharmaceuticals. During the development of next generation delivery system some points should be considered, which are as follows: (i) simplicity of the drug and its delivery system: the drug should be easy for manufacturing, quality control, handling and comparatively low-cost. (ii) Safety problems should be minimal. No extra chemical entities should be used which may affect structural stability. (iii) Oral delivery is still a challenge for therapeutic proteins and genes due to their resistance to proteolysis. Further, researches should be more inclined toward this mode of delivery.

## 8. Concluding Remarks

In the age of molecular medicine, a number of protein and gene deliveries have been developed while exploring liposomes, PEGylation, HESylation, and nanoparticle based methods. Past two decades have witnessed the accessibility of commercially available therapeutic products of protein and gene with the different kinds of delivery system. The next generation state-of-the-art gene based and protein based therapies may also improve effectiveness or reduce toxicities. Recent progress in the past two decades, in the field of protein and gene delivery, shows promise and provides bright hopeful future to the patients.

## Figures and Tables

**Table 1:** Next generation therapeutic proteins or genes and their delivery system which are in the market or in clinical trial.

Brand name of therapeutic protein/gene	Therapeutic protein/gene	Indication	Remark	References
Oncaspar	Therapeutic protein (pegylated formulation of L-asparaginase; Pegaspargase)	Leukaemia	USFDA-approved in 1994	[50]

PEG-INTRON	Therapeutic protein (pegylated formulation of IFN-a2B; Peginterferon alfa-2b)	Hepatitis C as well as malignancies	USFDA-approved in January 2000	[51]

PEGASYS	Therapeutic protein (pegylated formulation of IFN-a2A; Peginterferon alfa-2A)	Hepatitis C	USFDA-approved in January 2001	[52]

Neulasta	Therapeutic protein (pegylated formulation of Granulocyte-colony stimulating factor (GCSF) and monomethoxypolyethylene glycol; Pegfilgrastim)	Neutropenia	USFDA-approved in January 2002	[53]

Mircera	Therapeutic protein (pegylated formulation of Erythropoietin (EPO); Epoetin beta-methoxy polyethylene glycol)	Anemia associated with kidney disease	USFDA-approved in January 2007	[54]

— (No brand name available)	Therapeutic gene (RNAi therapeutics delivery of ALN-PCS02 using SNALP liposome)	Hypercholesterolemia	Clinical trial	[55]

Glybera	Therapeutic gene (alipogene tiparvovec used adeno-associated virus serotype 1 (AAV1) viral vector delivery)	Familial lipoprotein lipase deficiency (LPLD, synonym: type I hyperlipidaemia).	First gene-therapy medicine and approved by all 27 European Union member states	[56]
